# PPAR Gamma and Hepatic Stellate Cells

**DOI:** 10.1186/1476-5926-2-S1-S7

**Published:** 2004-01-14

**Authors:** Saswati Hazra, Takeo Miyahara, Richard A Rippe, Hidekazu Tsukamoto

**Affiliations:** 1 Department of Pathology, Keck School of Medicine of the University of Southern California, Los Angeles, California 90033', USA; 2 Department of Medicine, University of North Carolina, Chapel Hill, North Carolina 27599-7038, USA; 3 Greater Los Angeles VA Health Care System, Los Angeles, CA 90073, USA

## Abstract

Activation of Hepatic stellate cells (HSC) in fibrogenesis involves distinct morphological and biochemical changes. This activation requires the coordinated changes in activity of several transcription factors. Peroxisome proliferator-activated receptor gamma (PPAR gamma) is one such factor whose activity is decreased in activated HSC. PPAR gamma ligands suppress several markers of HSC activation such as expression of collagen and alpha smooth muscle actin (alpha-SMA), cell proliferation and migration. Expression of PPAR gamma, per se, also inhibits HSC activation. These findings support the role of PPAR gamma in reversion of activated HSC toward their quiescent state.

## Introduction

Hepatic stellate cells (HSC) are vitamin A storing, perisinusoidal pericytes that undergo phenotypic changes characterized as "myofibroblastic activation" during liver fibrogenesis. This activation process involves morphological and biochemical changes. To understand the molecular basis of the activation, many laboratories have characterized alterations in specific gene regulation and intracellular signaling that may confer phenotypic changes of activation. Depletion of retinoids is one of the key features in HSC activation. The contents of all-trans and 9-cis retinoic acids (biologically active metabolites of vitamin A), are also decreased in activated HSC. RAR and RXR, the nuclear receptors for these retinoic acids are also reduced in activated HSC [1]. RXR heterodimerizes with the members of the steroid/thyroid hormone receptor superfamily, such as vitamin D receptor, peroxisome proliferator-activated receptor (PPAR) and liver X receptor (LXR). PPAR gamma is a ligand dependent transcription factor that is predominantly expressed in adipose tissue and plays an important role in adipogenesis, fat and energy metabolism. There are at least three isoforms of PPAR gamma (gamma 1, gamma 2, gamma 3). PPAR gamma 2 is predominantly expressed in fat tissue while PPAR gamma 1 is found in various organs including liver. Activated PPAR gamma regulates transcription of several genes after binding to a specific PPAR response element, PPRE, a hexameric direct repeat (TGACCT) separated by a single nucleotide. This article reviews the considerable advances that have recently been made for the regulatory role of PPAR gamma in activation of HSC.

## Expression of PPAR Gamma in HSC

**Figure 1:**
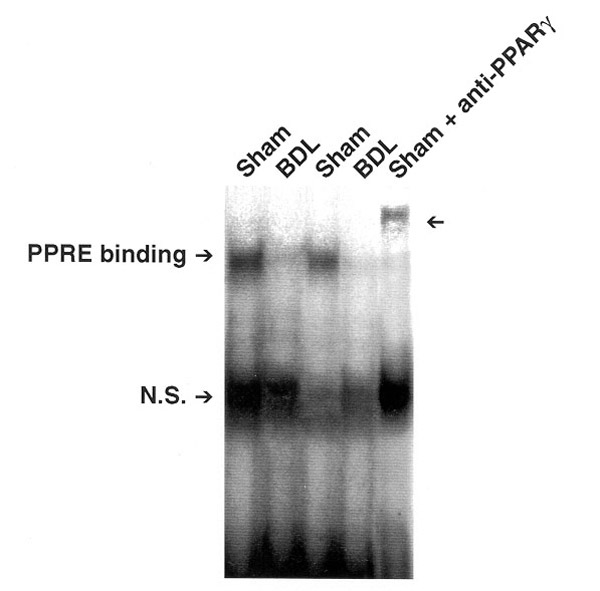
PPRE binding is diminished in HSC from BDL compared to sham animals.

The transcription factors such as NF-kappa B, AP1 and KRUPPEL like transcription factor are all associated with the activated phenotype of HSC. However, the expression and activity of PPAR gamma are suppressed in activated HSC. In fact, three independent studies demonstrated that PPAR gamma activity was reduced in cultured activated human and rat HSC [2-4]. Our *in vivo *studies [4] also revealed a decrease in the expression of PPAR gamma in HSC from a bile-duct ligated rat model (HSC-BDL) as compared to HSC from sham operated normal rat (HSC-sham) (Fig. 1).

**Figure 2:**
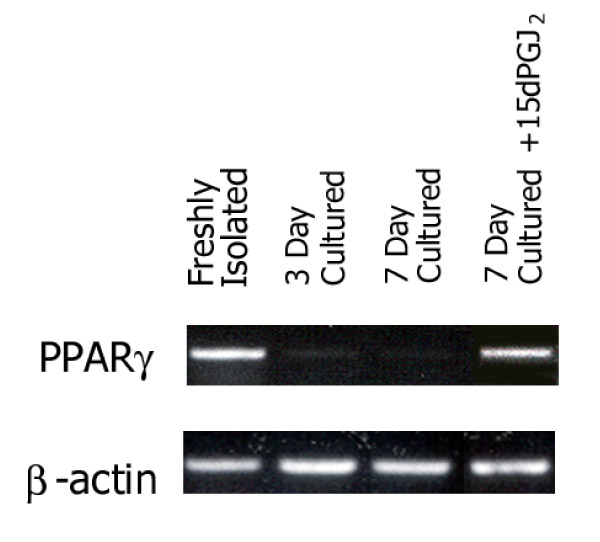
Culture activation of HSC rapidly decreases PPAR gamma expression at 3 and 7 days in culture. 15dPGJ2 treatment for 18 h to 7 day cultured HSC restores PPAR gamma expression (last lane).

To directly assess the binding of PPAR gamma to PPRE, electrophoretic mobility shift assay (EMSA) was performed using the ARE7 probe (a PPRE element of aP2 gene). PPRE binding of nuclear extracts from HSC-BDL was decreased compared to that in HSC-sham. The specificity of binding was supported by supershift assay using anti-PPAR gamma antibodies. Interestingly, the treatment of culture-activated HSC with a natural ligand for PPAR gamma (15dPGJ2) caused reappearance of PPAR gamma mRNA (Fig. 2).

## Effects of PPAR Gamma Ligands on Activation Markers of HSC

To assess the relationship between PPAR gamma and activation of HSC, the effects of PPAR gamma ligands on HSC activation markers have been addressed. Miyahara *et. al *demonstrated that 15dPGJ2 and BRL49653 (rosiglitazone) suppressed DNA synthesis by 40–50% in cultured activated HSC [4]. Galli *et al. *and Marra *et al. *also independently showed that PDGF stimulated proliferation of human HSC was inhibited by 15dPGJ2, ciglitazone and troglitazone [2,3]. Collagen production, another fundamental parameter of HSC activation, was also inhibited by 15dPGJ2 [4]. This inhibition was blocked with a PPAR gamma antagonist (GW9662). Further, alpha-SMA expression, PDGF induced HSC migration and cytokine induced MCP-1 expression, other features of HSC activation, were also inhibited by thiazolinedione (TZD) and 15dPGJ2 [4]. Finally, the treatment with TZD was shown to ameliorate liver fibrosis in both toxic and cholestatic models [5]. All these findings support a notion that PPAR gamma ligand treatment inhibits HSC activation and liver fibrosis. However, a recent study by Chawla, *et al., *demonstrated that so called "PPAR gamma ligands"(15dPGJ2 and TZD) rendered some of known biological effects via PPAR gamma independent manner [6]. This raises a question as to whether PPAR gamma itself is a true effector molecule for the previously observed effects of PPAR gamma ligands on HSC.

## Special Effects of PPAR Gamma on HSC Activation

To demonstrate the effects of PPAR gamma, per se, Galli *et al. *[2] transfected cultured activated human HSC with a PPAR gamma expression vector. In this study, PDGF-stimulated proliferation of HSC was decreased with expressed PPAR gamma without ligand treatment. They further reported the inhibition of TGF beta 1-induced human alpha 2(I) procollagen promoter activity by transduced PPAR gamma. Our recent transfection results suggest that cultured activated HSC are depleted of PPAR gamma but not its endogenous ligands (unpublished data), raising an interesting question as to why these ligands are not driving PPAR gamma expression in these cells. We further tested the effect of adenoviral-mediated expression of PPAR gamma on activated HSC. By normalizing PPAR gamma expression in this manner in culture-activated HSC, activation markers such as TGF beta 1 and alpha 1(I) procollagen genes were clearly suppressed and the morphology of activated HSC was reverted toward the more quiescent phenotype [7].

## Conclusions

These findings from various laboratories demonstrate that activation of HSC is associated with depletion of PPAR gamma. Expression of PPAR gamma or addition of its ligands reverts characteristic biochemical and morphological changes of HSC activation suggesting the importance of PPAR gamma in the maintenance of the quiescent HSC phenotype.
